# Pancreatic islets: new discoveries for innovative treatments of diabetes?

**DOI:** 10.1007/s00125-026-06839-7

**Published:** 2026-08-21

**Authors:** Raphael Scharfmann, Arnaud Zaldumbide

**Affiliations:** 1 Institut Cochin, Université Paris Cité Inserm, Paris, France; 2 Department of Cell and Chemical Biology, Leiden University Medical Center, Leiden, the Netherlands

**Keywords:** Beta cell biology, Imaging, Islet structure, Review, Type 1 diabetes

## Abstract

Pancreatic beta cells are dedicated factories in the production, storage and secretion of impressive amounts of insulin. In the human pancreas, beta cells are found in clusters known as islets of Langerhans which vary in cell quantity and endocrine cell types, including glucagon-producing alpha cells and somatostatin-producing delta cells. Major progress has been made in recent years in understanding beta cell physiology and pathology, with a particular focus on the mechanisms of beta cell destruction in type 1 diabetes and potential approaches for restoring beta cell function. In this review, we have selected recent examples that highlight the progress made in understanding human islets and beta cell biology. Specifically, we (1) concentrated on new discoveries in beta cells, both within and outside of the islets of Langerhans; (2) evaluated whether more understanding of basic beta cell biology is needed; (3) discussed whether more tools are needed to provide answers to our key questions; as an example, we highlighted the progress made in the development of human beta cell lines and in the imaging tools for studying human beta cells; (4) questioned why there is still a gap in knowledge regarding beta cell destruction in type 1 diabetes, despite hundreds of thousands of published studies. We conclude with our vision of beta and islet cell biology.

**Graphical Abstract:**

**Figure:**
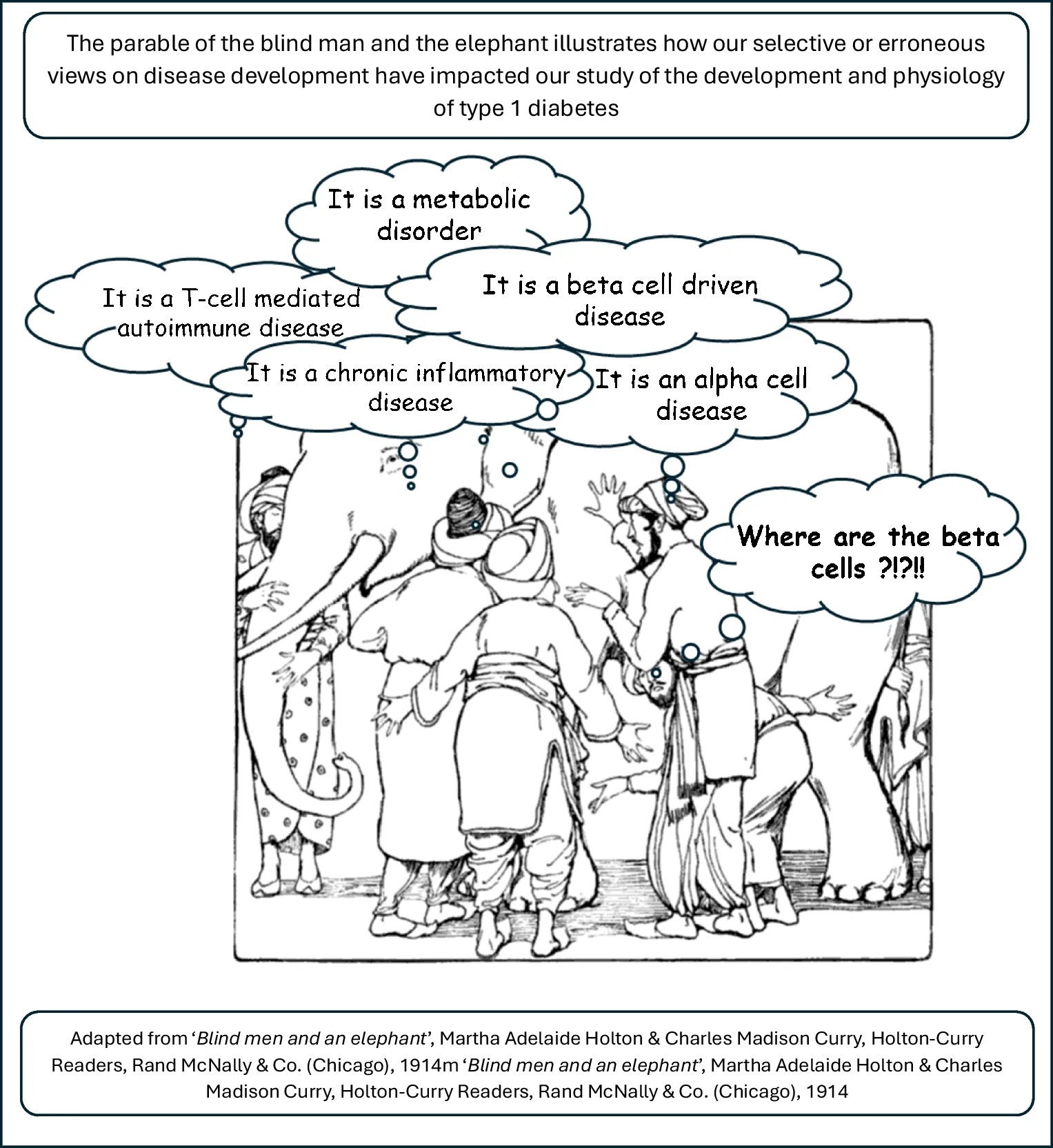

**Supplementary Information:**

The online version contains a slide of the figure for download available at https://doi.org/10.1007/s00125-026-06839-7.

## Introduction

As scientists working in the field of islet cell biology, our main task is to increase knowledge. If robustly performed, our work should give rise to discoveries that can treat or even cure pathologies such as diabetes. Major progress has been made in past decades in the understanding of insulin-producing beta cell development within the islets of Langerhans. This valuable information gave rise to a number of achievements. For example, numerous clinical trials have surfaced thanks to the new-found knowledge of how the immune system destroys beta cells and the developed ability to produce beta cells from stem cells. However, our progress is not sufficient, as it is evident that we lack key information about pancreatic islets in physiological and pathological conditions. Such knowledge is needed to protect insulin-producing beta cells against cell death in type 1 diabetes and to compensate for insufficient functional beta cell mass in type 2 diabetes. In this review we examine several points that we believe are central to understanding pancreatic islet and insulin-producing beta cell physiological function in normal health and in diabetes. We also describe some outside-of-the-box examples that gave rise to unexpectedly original results. We conclude with our vision of the importance of protecting fundamental academic research.

## New discoveries of beta cells within and outside of islets of Langerhans

Islets of Langerhans are small micro-organs dispersed within the pancreatic gland and composed of different endocrine cell types (beta, alpha and delta cells), endothelial cells, fibroblasts and immune cells. Pancreases from adult mice and rats used in laboratories contain 10^3^ to 10^4^ islets of quite similar size (±1000 cells per islet). Within every rodent islet, endocrine cells are well organised with a core of beta cells (±80% of the cells) surrounded by alpha (±15%) and delta (±5%) cells. This classically described cell arrangement [1] has recently been challenged by a study demonstrating that the number of non-beta endocrine cells (~20%) is insufficient to fully cover the beta cell core of an islet [2]. Furthermore, this arrangement is not conserved during evolution, leading to more stochastic rearrangement in humans and possibly reflecting an optimal reorganisation to adapt to specific constraints. In humans, it has long been known through the staining of 2D pancreatic sections that alpha and delta cells are scattered throughout the islets, which is different from what is observed in laboratory rodents [3, 4]. Our first naive question is: Do islets from rodent lab models reflect the same cellular arrangement found in rodents dwelling in natural wild habitats?

In years prior, immunostaining of human pancreatic sections revealed a high frequency of small insulin-positive cell clusters of 1–3 cells that were not associated with other pancreatic endocrine cell types. Quantitative analyses revealed that these small cell clusters represent up to 15% of all beta cells [5]. The large quantity of small beta cell clusters in the human pancreas was recently validated through 3D [6, 7] and 2D imaging [8]. A 3D analysis found that half of the human insulin-expressing islets are virtually devoid of glucagon-producing alpha cells [6]. This raises the question of whether there is a type of functional heterogeneity in terms of insulin secretion between beta cells clustered with alpha cells and islets solely composed of beta cells. However, so far, models developed to dissect the interplay between the islet composition and its function and assess the role of non-beta endocrine cells in insulin secretion have led to contradictory results. Neither the in vivo transgenic mice using a drug-induced selective alpha or delta cell ablation [9], where an extra-pancreatic GLP1 effect (from L cells) could not be ruled out, nor the in vitro study [10] using pseudo-islets formed upon cell dissociation, sorting and reaggregation which disrupt the islet structure and vascularisation appear to be ideal to address this question. While an apparent consensus is missing, a plethora of publications have illustrated the importance of alpha and delta cells in beta cell function (as outlined recently by M. Huising [11]). In this context, the contribution of the small beta-only clusters described above to the overall insulin secretion remains an open question.

Another recent discovery based on 2D and 3D insulin staining of human pancreases from control and type 1 diabetes donors indicates that small islets (extra-islet beta cells) are virtually absent in pancreases from type 1 diabetes donors, suggesting their preferential destruction by the immune system [7, 8]. Are these small islets actually destroyed beyond detection, and could they undergo a greater degree of destruction than larger islets? Or are they simply harder to detect? Rippa et al [7] indicate that in type 1 diabetes donor pancreases, residual INS^+^ cells are predominantly preserved in large INS^+^/GCG^+^ islets. Are INS^+^ cells present in large islets containing alpha and delta cells different from isolated beta cells? Or could it be that GCG^+^ islets protect beta cells against destruction? Spatial omics could provide answers to these questions.

## Do we need more knowledge of fundamental beta cell biology?

Human beta cells are long-lived cells, as supported by mathematical modelling of lipofuscin accumulation (a feature of ageing in post-mitotic cells) [12], in vivo thymidine analogue incorporation and radiocarbon dating in human beta cells [13]. These small insulin-producing factories (±10^9^ beta cells per human pancreas) are impressively robust and efficient producers that seem to live >30 years. One study calculated that the endoplasmic reticulum of a single beta cell produces close to 1 million molecules of insulin per minute [14], while a mathematical calculation by M. Solimena concluded values 2–10 times lower (M. Solimena, TU Dresden, Germany, personal communication) using the following parameters: (1) a beta cell contains 5000–10,000 secretory granules/cell; (2) each secretory granule contains 200,000–400,000 insulin molecules; and (3) secretory granules have a half-life of 4–5 days. In either model, beta cell insulin production nonetheless remains impressive. Moreover, it was found years ago that human islets can be kept in culture and remain functional for up to 2 years [15]. This supports that there is an extremely low human beta cell turnover in adult life. This could be different from what is observed in mice, where beta cell proliferation has been observed in a recurrent fashion; however, the vast majority of these data have been obtained from juvenile mice and data from older mice indicate a major decline in beta cell proliferation [16, 17]. It is thus not fully evident whether adult beta cells proliferate more in mice than in humans. These beta cell parameters constitute vital information as there are large debates over these topics. For example, a current scientific endeavour is to regenerate beta cell mass in individuals with type 1 and type 2 diabetes. Part of the scientific community views human beta cells as post-mitotic and with the inability to regenerate during adult life [18–20]. Others in the community believe that beta cells do regenerate during the adult human life. As an example, harmine, used as a DYRK1A inhibitor, was discovered to increase human beta cell mass (in reconstructed models) by activating beta cell proliferation [21]. More recent data suggest a more complex pathway where harmine increases the proliferation of alpha cells which then transdifferentiate into beta cells [22]. This concept of alpha to beta cell transformation had been highlighted in a mouse study 10 years ago, in a seminal publication where γ-aminobutyric acid (GABA), a major inhibitory neurotransmitter, induced an impressive alpha to beta cell conversion. This phenomenon also occurred in rat and human islets [23]. However, the follow-up conducted by Novo Nordisk had major difficulties in reproducing the published mouse data [24] and the clinical trial (ClinicalTrials.gov registration no. NCT03721991) concluded that long-term GABA treatment fails to regain beta cell function in longstanding type 1 diabetes [25]. Interestingly, this team working on alpha to beta conversion restructured the hypothesis and continued to work on the regeneration theory from the perspective of proliferation rather than transdifferentiation. They describe R-Spondin, a main agonist of the Wnt/β-catenin pathway for human beta cell amplification, as a key player in proliferation [26]. However, we see that there is some evidence in favour of the concept of poor beta cell turnover in the human adult pancreas. This is based on observation of several sections of different pancreases that did not show any sign of beta cell proliferation (mitotic figures or Ki67^+^ cells). Moreover, the presence of cells co-expressing INS and GCG was far from evident. Thus, rare events (possibly under the detection rate) of beta cell proliferation and alpha to beta cell transdifferentiation may accumulate over decades of life. But are those events clinically meaningful for individuals with type 1 or type 2 diabetes? This idea is further supported by a recent review [27] in which the authors noted that human adult beta cell turnover is limited (even under the best conditions) and it would take years of continuous stimulation to restore physiological beta cell mass. It is clear that fundamental knowledge is still lacking and that innovative tools need to be developed to help uncover this knowledge. Constructive discussions around bottles of French wine are also required for progress in unravelling this mysterious topic.

## Do we need more tools to get answers to our questions?

### Human beta cell lines as a first example

It is evident that the development of innovative tools is beneficial for the generation of new knowledge in biology, including islet biology. The development of human beta cell lines represented one of these tools. Between 1990 and the early 2000s, our Inserm team started developing in vitro models based on fetal rat pancreatic epithelium cultures containing progenitors and lacking surrounding mesenchyme. Using this simple model of organotypic culture (which some would now name organoids), we proposed that pancreatic progenitors efficiently differentiate into endocrine cells through a default pathway [28]. At that time, we had the opportunity to join forces with a business angel which gave us the money to select and perform our ideal scientific project design. We had full freedom and reign, a dream for all scientists. We used our expertise on pancreatic development and islet biology to make human beta cell lines as a tool to study human beta cell biology. At the time, a number of teams had tried to establish a human beta cell line but failed [29, 30]. We became successful in developing a multistep protocol by building a team comprising colleagues (with whom we formed great friendships) and outstanding engineers. Our strategy was based on targeted oncogenesis in human fetal pancreases using lentiviral vectors to express the oncogene products SV40T and hTERT under the control of the well-characterised and strongly beta cell-specific rat insulin promoter [31]. We performed our first experiments with human fetal pancreatic fragments cultured in the above-described model of rodent pancreatic organotypic culture, but we failed to amplify human beta cells. We came up with a creative solution: in those days, we had developed expertise in transplanting human fetal pancreases into immune-deficient mice, which we used as an incubator to induce pancreatic cell growth and differentiation [32]. We shifted our approach and used this in vivo incubator in place of tissue culture. This allowed us to differentiate targeted cells into beta cells and to amplify them. In 2005, an article [33] was published by an international team that had succeeded in generating a functional human beta cell line, named NAKT-15. We celebrated the success for the community, although we were envious as we could not be the first to achieve it. Nonetheless, we have been lucky to be able to carry our project this far, and we have made great progress (the NAKT-15 cell line has been used in a limited number of original publications [according to Google Scholar]). We eventually succeeded in generating a family of human beta cell lines (EndoC-BH) [18, 34, 35] which have been used in hundreds of original publications (https://www.humanbetacelllines.com/). How did we succeed? We had synergistic expertise in basic biology of pancreas development, rodent and human islets, lentiviral vector development and transplantation in immune-compromised mice. Other key factors were time, patience, friendship and freedom of scientific direction. Noteworthily, we published our first paper on human beta cell lines 12 years after the start of the programme, which is quite unusual in both academic and industrial biology. This emphasises our patience and dedication in developing a robust method. In 2004, we were encouraged by our business angel to create a company, EndoCells, now named Human Cell Design. We thus may be considered ‘Pasteur’s quadrant researchers’, with the potential to bring novelty and impact to publishing and patenting [36].

### A second example is tools for imaging and targeting beta cells

Live visualisations or living snapshots of hepatocytes in the liver, glomeruli in the kidney, specific brain regions and heart using imaging tools and biopsies have allowed for the development of an evolved toolbox, which provides deeper insights into molecular mechanisms driving disease development while also promoting target-specific drug discovery. In contrast, the islets of the pancreas represent a million dispersed black boxes. The human pancreas cannot be biopsied (the DiViD study being a unique case [37]) and islets can be neither visualised nor quantified in living people. We use the term ‘functional beta cell mass’ as an imprecise definition for beta cell mass and function. Some years ago, the European IMIDIA consortium composed of academic and industrial partners joined forces to progress the imaging of human beta cells, and they summarised their work in a review titled ‘Pancreatic β-cell imaging in humans: fiction or option?’ [38]. Ten years later, we have the feeling that it remains a fiction despite some progress. For example, novel beta cell surface biomarkers, such as DPP6 and FXYD2γ, have been identified and strategies for first-in-man testing have been proposed [39]. Moreover, the use of labelled ligands in an attempt to quantify human beta cell mass was developed based on the fact that GLP1R is highly enriched in beta cells when compared with other pancreatic cell types. Such labelled ligands were efficient in detecting human islets in the liver of transplanted individuals [40]. Time will tell whether this non-invasive approach can be further developed to measure residual beta cell mass during type 1 diabetes progression. Interestingly, others have demonstrated that in mice, a single subcutaneous injection of antisense oligonucleotides against the transcription factor FOXO1 conjugated to a ligand of the GLP1R was sufficient to decrease FOXO1 mRNA by more than 50% within islets [41]. This is a first proof of concept that pancreatic beta cells can be targeted in vivo in mice. A second proof of concept is the identification of RNA aptamers that selectively recognise mouse and human beta cells, which were used to measure beta cell mass of human islet-transplanted mice in a semi-quantitative manner [42]. Other strategies have been investigated to extrapolate the pancreatic beta cell mass or the beta cell decay in type 1 diabetes using fluid biomarkers. Besides metabolic markers (stimulated C-peptide or HbA_1c_), a few beta cell by-products such as circulating miRNAs (e.g. miR-375) and unmethylated insulin DNA have been proposed to accurately reflect beta cell mass in the context of islet transplantation [43, 44]. Yet, it remains debatable whether or not the large individual and disease heterogeneity may compromise their use in evaluating the residual beta cell mass in the context of type 1 diabetes. Here again, time will tell.

In conclusion, we are slowly progressing from fiction to feasible options, and this progress should continue, possibly by bringing physicists interested in imaging and targeting human pancreatic beta cells to the discussion table.

## Why don’t we know everything about beta cell destruction in type 1 diabetes yet?

**Fig. 1:**
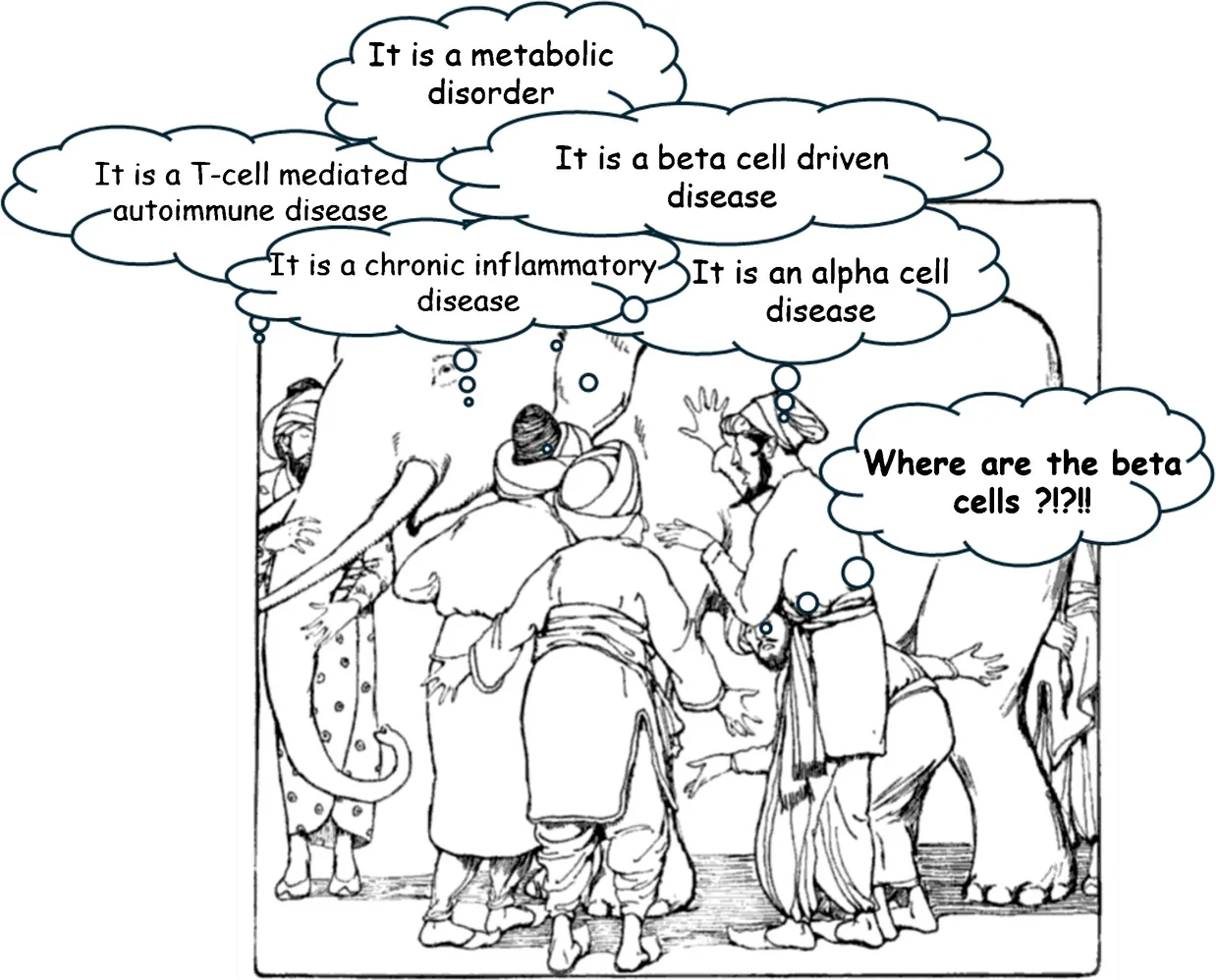
‘Blind men and an elephant’. This parable illustrates some remaining questions and our selective or erroneous views on disease development, islet organisation or beta cell biology that have impacted our conclusions. We may not be blind but the monocentric view of type 1 diabetes focusing solely on the immune system (defect in thymic education) has considerably influenced our therapeutical strategies. The disappointing outcomes of some of those trials underscore other layers of complexity as well as an important role for beta cells in driving autoimmunity and the possible participation of other endocrine cells in the pathology. Adapted from ‘*Blind men and an elephant*’, Martha Adelaide Holton & Charles Madison Curry, Holton-Curry Readers, Rand McNally & Co. (Chicago), 1914. This figure is available as a downloadable slide

Some years ago, we wrote a commentary on islet pancreatic endocrinogenesis entitled ‘I have all the answers, who has the questions?’ [45]. We do believe that in type 1 diabetes the main questions to be answered lie at the root of the disease. Such questions include: How can specific destruction of the beta cells be explained? How are the neighbouring alpha cells or delta cells, which are in close contact, not targeted by the infiltrating immune cells? Several studies and reviews have been written by colleagues to solve this identity dilemma and to determine what makes beta cells different from the other endocrine cells [46]. With the development of single cell omics technologies and the efforts of the scientific community to compile all omics datasets through the formation of large consortia (e.g. HPAP, Pancreasatlas, ESPACE, Human Cell Atlas [HCA]), we have learned that endocrine cells differ in shades of grey, as illustrated by the alpha–beta cell plasticity made possible by modulating only two transcription factors (e.g. PDX1 and MafA) [47]. We have also identified their respective responses to pathophysiological conditions and subtle differences in their specific transcriptional signatures, which suggest a more elaborate antioxidant system, a better adaptive stress response or higher expression of anti-apoptotic proteins that could confer upon alpha cells a selective advantage over beta cells in surviving inflammation and immune attack [46]. However, are those differences sufficient on their own to explain the specific alteration of the beta cell mass in type 1 diabetes? Probably not. These transcriptomic analyses have made it evident that the endocrine cell subset clustering was mainly dependent on the expression of the pancreatic hormones encoding genes [48]. Hence, by focusing on details, we forgot the elephant in the room (Fig. 1). What if insulin, whose expression is restricted to beta cells, in contrast to glucagon- or somatostatin-related products, and thereby restricts peripheral tolerance, turned out to be the Achilles heel of islets and beta cells? In other words, would mis-expression of insulin in alpha cells, delta cells or other endocrine cells be sufficient to trigger their destruction in an in vivo type 1 diabetes model? Several studies have already identified insulin as the first and main autoantigen in type 1 diabetes [49, 50], connecting beta cell function (or beta cell hyperactivity) with autoimmunity. These observations have laid the groundwork for investigating the impact of environment transcriptional and translational fidelity in order to identify potential insulin-derived neoantigens for which central tolerance may be lacking. Indeed, referring to the previously cited calculations illustrating beta cell insulin production capacity at high demand (e.g. >100,000 molecules of insulin per minute), rare ‘mistakes’ during insulin production would represent a non-negligeable amount of protein that could be recognised as ‘non-self’ by the immune surveillance machinery. A small percentage from a huge number can give rise to a big figure! The discovery of the defective ribosomal product of insulin (DRIP), and the identification of CD8 T cells directed against this aberrant insulin product in the blood but also infiltrating the islets of individuals with type 1 diabetes, illustrate that beta cells are not passive victims of the immune system, but that they are actively contributing to their own destruction [51]. The discovery of insulin DRIP and the identification of circulating specific T cells in PBMCs of individuals with type 1 diabetes and their participation in beta cell destruction also challenged the view that autoimmunity derived solely from impaired thymic education. In this new model, beta cells would be destroyed by a mechanism that we have identified as comparable to classical antitumour responses, whereby the immune system has been trained to survey for dysfunctional cells in which non-native errors (transcriptional or translational) or modified antigens (post-translational modifications or proteasome-generated hybrid peptides) have accumulated, as also observed in other autoimmune diseases [52]. Yet, postulating that beta cell autoimmunity is tightly associated with beta cell function implies that molecules associated with insulin production or secretion (e.g. insulin granules or the beta cell-specific peptide islet amyloid pancreatic polypeptide [IAPP], co-secreted with insulin) also participate in the increased visibility of the cells to the immune system [53]. This association between beta cell function and immunogenicity is paradoxical and indicates that beta cell protection could be at the cost of beta cell function. ‘Less is more’? This acceleration hypothesis, connecting the rising demand in insulin with autoimmunity, and proposed over 30 years ago [54], is supported by recent epidemiological studies [55] and the presence of sleeping beta cells [56] (inactive and immune-protected cells) detected in pancreas sections of newly diagnosed participants from the DiViD study or the nPOD programme. This echoes the results from mouse studies where glucose challenge was shown to induce deleterious effects on islets through the induction of thioredoxin-interacting protein (TXNIP), a key regulator of beta cell apoptosis [57]. The discovery that L-type calcium channel blockers, inhibiting insulin secretion, reduce glucose-induced TXNIP expression and downstream deleterious effects [58] provided the rational for evaluating the role of verapamil in delaying type 1 diabetes progression. The successful Ver-A-T1D trial outcomes, showing better preservation of the beta cell function and a slower rate of beta cell loss (to be validated in phase 3) [59], further strengthen this hypothesis and demonstrate that early intervention aimed at lowering beta cell activity might be beneficial in preventing their destruction by the immune system. We must, however, be humble. We cannot exclude the fact that verapamil does not act on beta cells to decrease TXNIP levels in the human trial, but instead acts on another target, such as immune cells, to indirectly protect beta cells. To reiterate, more tools are needed for progress. Altogether, these studies provide a metabolic component to type 1 diabetes, which to an extent bridges type 1 and type 2 diabetes pathologies. While we can all agree that type 1 and type 2 diabetes have a different aetiology and genetic susceptibility, we might also consider that they may reflect two sides of the same coin and share beta cell stress, mitochondrial dysfunction, impaired insulin production and innate immune cell infiltration in the initial phases of the diseases [60, 61]. Can we understand type 1 diabetes pathology better to prevent or delay beta cell destruction through the study of type 2 diabetes? Possibly, yes. A series of ongoing preclinical trials aimed at repurposing type 2 diabetes drugs for the treatment of individuals with type 1 diabetes may support this concept. These approaches focus on improving glycaemic management, protecting beta cells or reducing beta cell stress (imposed by insulin demand and production) using SGLT2 inhibitors, GLP-1 receptor agonists, DPP-4 inhibitors, lifestyle interventions or ketogenic diets [62–64]. However, linking beta cell immunogenicity to beta cell function could pose a problem for future regenerative therapies aimed at producing highly functional stem-cell-derived beta cells. Do we want to generate ‘super beta cells’ with high insulin production capacity? Or more beta cells with reduced potential but that are less visible to the immune system?

Another point of concern is whether, within islets, the other endocrine cells, despite not being attacked by the immune system, are merely silent witnesses to the beta cell destruction. While the role of alpha cells in beta cell function is well described, in particular by the stimulatory effect of glucagon on insulin secretion [65], the crosstalk between alpha and beta cells during type 1 diabetes pathogenesis is less clear. Unger’s pioneering work in the 1970s [66] introduced the concept of a glucagonocentric hypothesis, presenting type 1 diabetes as a ‘bihormonal disease’, in which alpha cells are also dysfunctional, a concept further supported by more recent work [67, 68]. Was this a cause or consequence of the lack of insulin paracrine effect [67]? To further complicate matters, the direct paracrine effect remains under debate [69, 70]. In light of our recent work showing the increased expression of TXNIP observed in beta chimeric mouse islets depleted of alpha cells [10], as well as the numerous studies supporting the effect of both glucagon and GLP1 on beta cell function and survival [71–76], the question is certainly worth asking, and this old hypothesis may need to be revisited. However, we may not have all the tools to study the complex crosstalk between alpha and beta cells, or even further between the beta cells and other endocrine cells. Considerable efforts have been invested in generating alpha and delta cell lines, using an approach that had been successfully implemented for the EndoC-BH cells (see above), but without published results. Should we go back to the drawing board and use vectors with different cell-type-specific promoters to drive the SV40/hTERT cassette for immortalising human fetal cells? Create endocrine lines using CRISPR technology? Or develop novel differentiation protocols focusing on generating alpha or delta cells from pluripotent stem cells? This last approach was recently explored by adjusting the classical beta cell differentiation protocol through chromatin remodelling at the pancreatic endocrine progenitor stage to generate non-beta endocrine cells [77]. This represents a major opportunity not only to better assess the intra-islet communication and better understand the complex crosstalk that precedes immune cell infiltration, but also to prepare the next-generation iPSC-derived islet product for the achievement of precise blood glucose management in individuals with type 1 diabetes [78].

## Conclusion

By progressively increasing knowledge through the development of innovative tools, significant progress has been made in islet biology, physiology and diabetes, including some positive clinical trials. But more needs to be done, which requires time, patience, outside-of-the-box thinking, friendship and bringing curious naive scientists on board. We are lucky to work as researchers in the field of islet biology with a lot of freedom and really hope it will be the same for the future generation. To paraphrase Pierre Dac, the French humourist: the future lies ahead of us, and we will have it behind us every time we turn around.

## Supplementary Information

Below is the link to the electronic supplementary material.

Figure slide (PPTX 306 KB)

## Abbreviations

DRIP: Defective ribosomal product of insulin
GABA: γ-Aminobutyric acid
TXNIP: Thioredoxin-interacting protein
